# Assessing the Biological Mechanisms Linking Smoking Behavior and Cognitive Function: A Mediation Analysis of Untargeted Metabolomics

**DOI:** 10.3390/metabo13111154

**Published:** 2023-11-16

**Authors:** Jerome J. Choi, Rebecca L. Koscik, Erin M. Jonaitis, Daniel J. Panyard, Autumn R. Morrow, Sterling C. Johnson, Corinne D. Engelman, Lauren L. Schmitz

**Affiliations:** 1 Department of Population Health Sciences, School of Medicine and Public Health, University of Wisconsin-Madison, Madison, WI 53726, USA; choi267@wisc.edu (J.J.C.); armorrow@bwh.harvard.edu (A.R.M.); 2 Wisconsin Alzheimer’s Institute, School of Medicine and Public Health, University of Wisconsin-Madison, Madison, WI 53726, USA; rekoscik@wisc.edu (R.L.K.); jonaitis@wisc.edu (E.M.J.);; 3 Wisconsin Alzheimer’s Disease Research Center, University of Wisconsin-Madison, Madison, WI 53792, USA; 4 Department of Genetics, School of Medicine, Stanford University, Palo Alto, CA 94305, USA; dpanyard@stanford.edu; 5 William S. Middleton Memorial Veterans Hospital, Middleton, WI 53705, USA; 6 Department of Medicine, School of Medicine and Public Health, University of Wisconsin-Madison, Madison, WI 53792, USA; 7 La Follette School of Public Affairs, University of Wisconsin-Madison, Madison, WI 53706, USA; llschmitz@wisc.edu

**Keywords:** smoking, Alzheimer’s disease, mediation analysis, metabolomics, cognitive function

## Abstract

(1) Smoking is the most significant preventable health hazard in the modern world. It increases the risk of vascular problems, which are also risk factors for dementia. In addition, toxins in cigarettes increase oxidative stress and inflammation, which have both been linked to the development of Alzheimer’s disease and related dementias (ADRD). This study identified potential mechanisms of the smoking–cognitive function relationship using metabolomics data from the longitudinal Wisconsin Registry for Alzheimer’s Prevention (WRAP). (2) 1266 WRAP participants were included to assess the association between smoking status and four cognitive composite scores. Next, untargeted metabolomic data were used to assess the relationships between smoking and metabolites. Metabolites significantly associated with smoking were then tested for association with cognitive composite scores. Total effect models and mediation models were used to explore the role of metabolites in smoking-cognitive function pathways. (3) Plasma N-acetylneuraminate was associated with smoking status Preclinical Alzheimer Cognitive Composite 3 (PACC3) and Immediate Learning (IMM). N-acetylneuraminate mediated 12% of the smoking-PACC3 relationship and 13% of the smoking-IMM relationship. (4) These findings provide links between previous studies that can enhance our understanding of potential biological pathways between smoking and cognitive function.

## 1. Introduction

Smoking is the most significant preventable health hazard in the modern world [1], and it is associated with many risk factors known to impact health, with numerous clinical endpoints [2]. There is strong evidence that smoking can increase the risk of developing dementia, a general term for a loss of cognitive function that is severe enough to interfere with daily living. Smoking increases the risk of vascular problems via strokes or minor bleeds in the brain, which are also risk factors for dementia [3]. In addition, toxins in cigarette smoke increase oxidative stress and inflammation, which have been linked to the development of a type of dementia, Alzheimer’s disease (AD) [3].

Recently, studies have investigated the role of metabolites, small molecule substrates, intermediates, and products of cell metabolism in cognitive function [4,5]. Untargeted metabolomics [6] can be used to measure a wide range of metabolites in fluid or tissue and can be influenced by genetics, environmental factors, aging, and disease [7]. Because they are the end product of upstream cellular processes, metabolites provide a downstream functional signature of the small molecule changes associated with a phenotype, which makes them especially useful for identifying therapeutic interventions [8]. Studies have examined associations between metabolites and cognitive function in late midlife [9,10,11,12,13,14], as well as associations between metabolites and behavioral risk factors, including smoking [15]. However, the mediating role that metabolites may play in the association between smoking and cognitive function has not been investigated. We sought to address this gap using mediation analysis to identify whether metabolites profiled from untargeted metabolomics in plasma and cerebrospinal fluid (CSF) are in the biological pathway between smoking and cognitive function. Identifying such metabolites may provide a better understanding of the mechanism linking smoking to cognitive decline. 

## 2. Materials and Methods

### 2.1. Data and Study Population

The Wisconsin Registry for Alzheimer’s Prevention (WRAP) was established in 2001 [16] and is a longitudinal observational cohort study of over 1500 individuals predominantly aged 40–65 at baseline; the sample is enriched for a parental history of probable AD, but enrolled participants have no prior diagnosis of dementia or evidence of dementia based on cognitive testing at baseline [17]. Up to two decades of serial cognitive data have been collected alongside genetic data, plasma, and, in a subset of participants, CSF. We used the May 2020 release of the WRAP data, which contained up to seven visits for 1561 participants. Since key variables such as smoking status and cognitive composite scores were available starting at the second visit and only a few participants had completed the visit seven follow-up assessment to date, we treated the second visit as baseline and excluded the seventh visit. There were 4680 observations for 1266 individuals with complete smoking, covariate, and cognitive composite score data and who remained free of dementia at visit 2.

### 2.2. Smoking Status

We derived a categorical variable for smoking status for never, former, and current smokers. The never smoker category was defined as participants who had never smoked. The former smoker category included participants who had reported ever smoking cigarettes but who had not smoked cigarettes in the past month. The current smoker category included participants who reported being ever smokers who also smoked in the past month. For the analyses, we coded smoking status as a numerical variable.

### 2.3. Cognitive Function

Cognitive function was evaluated with a global cognitive composite score, the 3-test Preclinical Alzheimer Cognitive Composite (PACC3) [18], and three domain-specific composite scores: Immediate Learning (IMM), Delayed Recall (DEL), and Executive Function (EXE) [19], as these measures have been found to outperform empirically derived composites or raw scores from single tests [20]. PACC3 consists of the Rey Auditory Verbal Learning Test (RAVLT) total trials 1–5, the Logical Memory subtest of the Wechsler Memory Scale-Revised (WMS-R LM) delayed recall, and the Digit Symbol Coding subtest of the Wechsler Abbreviated Intelligence Scale-Revised (WAIS-R). IMM consists of the RAVLT total trials 1–5, Wechsler Memory Scale–Revised Logical Memory subtest (WMS-R LM) immediate recall, and Brief Visuospatial Memory Test (BVMT-R) immediate recall. DEL consists of RAVLT long-delay free recall, WMS-R LM delayed recall, and BVMT-R delayed recall, and WMS-R LM delayed recall. EXE consists of Trail Making Test Part B total time to completion, Stroop Neuropsychological Screening Test color-word interference, and WAIS-R Digit Symbol Coding. As described previously [20], the composite scores were computed by first standardizing all contributing raw scores to a mean of 0 and a standard deviation (SD) of 1. If lower scores indicated better performance, the scores were multiplied by −1.

### 2.4. Covariates

Demographic characteristics including age, sex, race, and education were collected at baseline and included in both full and reduced models. Depressive symptoms, weekly alcohol consumption, and body mass index (BMI) were measured at each visit and controlled for in the full models because they are potential confounders that are associated with smoking behavior and cognitive outcomes [21,22,23]. Education was a dichotomous variable set equal to 1 if individuals earned at least a college degree and 0 otherwise. Depressive symptoms were measured using the Center for Epidemiologic Studies Depression Scale (CES-D) test scores. BMI was categorized into underweight, normal weight, overweight, and obese according to definitions from the Centers for Disease Control and Prevention.

### 2.5. Metabolomic Data Collection

The CSF and plasma collection and metabolomics analysis have been described in detail previously [24]. Briefly, CSF was collected via lumbar puncture (LP) in the morning after a 12-h fast. Blood for plasma samples was collected into ethylenediaminetetraacetic acid (EDTA) tubes. All samples were processed and stored at −80 °C until overnight shipment to Metabolon, Inc (Metabolon), Morrisville, NC 27560, where they remained frozen at −80 °C until analysis. Metabolon used Ultrahigh Performance Liquid Chromatography-Tandem Mass Spectrometry [25,26] to conduct an untargeted metabolomics analysis of the CSF and plasma samples.

### 2.6. Metabolomic Data Quality Control

Quality control was performed on the 412 CSF metabolites for 372 samples, including assessment of missingness, variation, and transformation. Thirteen metabolites were removed because of missingness >50%, one sample that had missingness >40% was removed, and nine low variance metabolites that did not satisfy the distribution of an interquartile range (IQR) >0 were removed. A log10 transformation was applied to each metabolite so they were more normally distributed. There were 390 CSF metabolites for 371 samples (169 individuals) after metabolite quality control.

Using the same quality control procedures for the 1275 plasma metabolites in 2500 samples, 112 metabolites were removed because of missingness >50%, none of the samples had missingness >40%, and 25 low variance metabolites that did not satisfy the distribution of an IQR >0 were removed. Similarly, log10 transformation was applied to each metabolite. There were 1138 plasma metabolites for 2500 samples (1236 individuals) after metabolite quality control.

After combining the smoking, covariate, cognitive composite score, and metabolomic data, there were 283 CSF samples for 166 individuals and 1871 plasma samples for 1188 individuals in three waves (visits 2, 3, and 4). The CSF samples were not always collected on the same day as the main visit (where cognitive testing, smoking assessment, covariates, and blood draw were obtained). In these cases, the CSF metabolite data were matched to the closest main WRAP visit by calculated age (derived by the WRAP data team from visit dates due to protected health information policies).

### 2.7. Statistical Mediation Analyses

**Figure 1:**
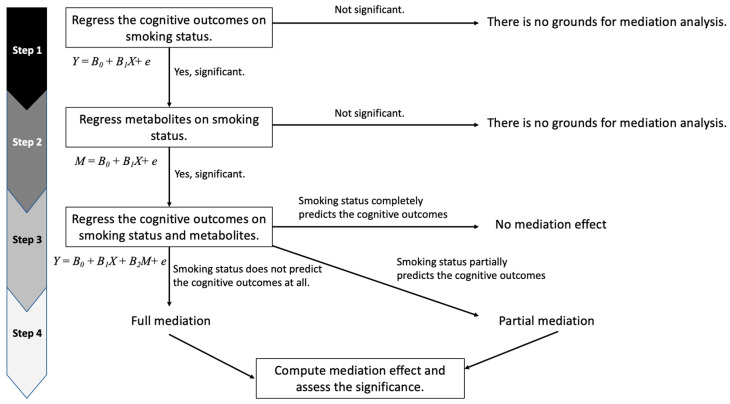
Mediation analysis flowchart. In Step 1, Y is a cognitive composite score, B_0_ is the intercept, B_1_ is the coefficient of smoking status, and X is smoking status. In Step 2, M is the level of a metabolite, B_0_ is the intercept, B_1_ is the coefficient of smoking status, and X is smoking status. In Step 3, Y is a cognitive composite score, B_0_ is the intercept, B_1_ is the coefficient of smoking status, B_2_ is the coefficient of a metabolite, X is smoking status, and M is the level of a metabolite.

To assess whether the smoking–cognitive function relationship is mediated by CSF or plasma metabolites, we broke down our analyses into four steps based on the product method (Figure 1) [27,28,29]. First, we tested whether smoking (exposure of interest) was associated with cognitive function (outcome). Second, we tested whether smoking was associated with each metabolite (mediator). Third, we tested whether each metabolite (mediator) associated with smoking was associated with the cognitive outcomes after adjusting for smoking. If a significant association between a metabolite and cognitive function remained after adjusting for smoking status in Step 3, we assessed whether the metabolite was completely or partially mediating the exposure–outcome relationship (Step 4).

Due to the longitudinal, multilevel structure of WRAP (i.e., individuals are nested within sibships across multiple visits), we used linear mixed models (LMM) implemented in R 3.6.1. to assess Steps 1–4 above for all four cognitive outcomes (PACC3, IMM, DEL, and EXE). Our reduced model included smoking status (reference category = never smoker), sex, race, education level, visit number minus two (practice effect; baseline was visit two), and linear and quadratic terms for age (centered to the mean). Random intercepts for family (except for models including CSF metabolites, which had very few related individuals) and individual and a random slope for age were included in the models to account for correlation between siblings and in repeated measures across individuals’ visits. Full models adjusted for additional confounders (CES-D, BMI, and weekly alcohol consumption) [30,31,32,33,34,35]. For each cognitive outcome, the model (reduced or full) with the best fit in Step 1 was used for subsequent steps.

In Step 2, adjustment for multiple hypothesis testing for models with each CSF and plasma metabolite were conducted using the false discovery rate (FDR) threshold of *p* < 0.05. The metabolites, as the outcomes, that were significantly associated with smoking status in Step 2 were used as predictors in the models in Step 3 and, among them, the metabolites that were also significantly associated with a cognitive outcome were retained for that outcome. Finally, in Step 4, mediation analyses were conducted to determine the mediation effects and direct effects in the pathway from smoking status to cognitive function. 95% confidence intervals (CIs) were generated using the distribution of product method and Monte Carlo method [36] using the R package *RMediation*.

## 3. Results

### 3.1. Sample Characteristics

The study sample consisted of 1266 participants with an average of 3.7 visits (median 4.0). Sample characteristics are shown in Table 1. The mean age at baseline was 58.5 years and the proportion of females (70.4%) was larger than males (29.6%). The majority of participants were white (94.8%), and 61.4% had college or graduate-level degrees. There were 86 (6.8%) current smokers, 469 (37.0%) former smokers, and 711 (56.2%) never smokers. The correlation between the four cognitive composite scores is shown in Supplementary Figure S1.

### 3.2. Mediation Analysis

#### 3.2.1. Step 1: Associations between Smoking Status and Cognitive Function

Results from the LMM analyses using full models, which adjusted for potential confounders, showed that being a current smoker was significantly associated with the four cognitive composite scores compared to nonsmokers (*p* ≤ 0.001; Table 2). Female sex, white race, a college or graduate degree, CES-D, weekly alcohol consumption and practice effects were significantly associated with higher cognitive function for all four composite scores (PACC3, IMM, DEL, and EXE). Having an underweight BMI was significantly associated with lower PACC3. Given the significant effects of the potential confounders, we used the full model in subsequent steps. Results from the reduced models are shown in Supplementary Material Table S1.

#### 3.2.2. Step 2: Associations between Smoking Status and Metabolomics

We used LMMs to test the association between smoking status and each metabolite. Among 390 CSF metabolites and 1138 plasma metabolites, 49 (12.6%) CSF metabolites and 630 (55.4%) plasma metabolites were significantly associated with smoking status (FDR < 0.05; Supplementary Table S2, Supplementary Figures S2 and S3).

#### 3.2.3. Step 3: Associations between Metabolites and Cognitive Function

After identifying 49 CSF metabolites and 630 plasma metabolites that were significantly associated with smoking status in Step 2, we used LMM to assess whether these metabolites were correlated with cognitive outcomes. We identified four plasma metabolites significantly associated with PACC3, IMM, and EXE (FDR < 0.05), including N-acetylneuraminate (NeuAc), androstenediol (3alpha, 17alpha) monosulfate (2), glycosyl-N palmitoyl-sphingosine (d18:1/16:0) (GlcCer), and metabolomic lactone sulfate (Table 3; full set of results are in Supplementary Table S3). None of the 49 CSF metabolites were significantly associated with cognitive function (FDR < 0.05). Step 4: Assessment of complete or partial mediation of the smoking status–cognitive function relationship.

Among those four plasma metabolites identified in Step 3, NeuAc and GlcCer were statistically significant mediators of the smoking–cognitive function relationship. For these two metabolites, total effect models were constructed to assess the effect of smoking status on cognitive outcomes. The effect of smoking status on cognitive function was then decomposed into the indirect effect (IE; the mediation effect) and the direct effect (DE) in the mediation models.

**Figure 2:**
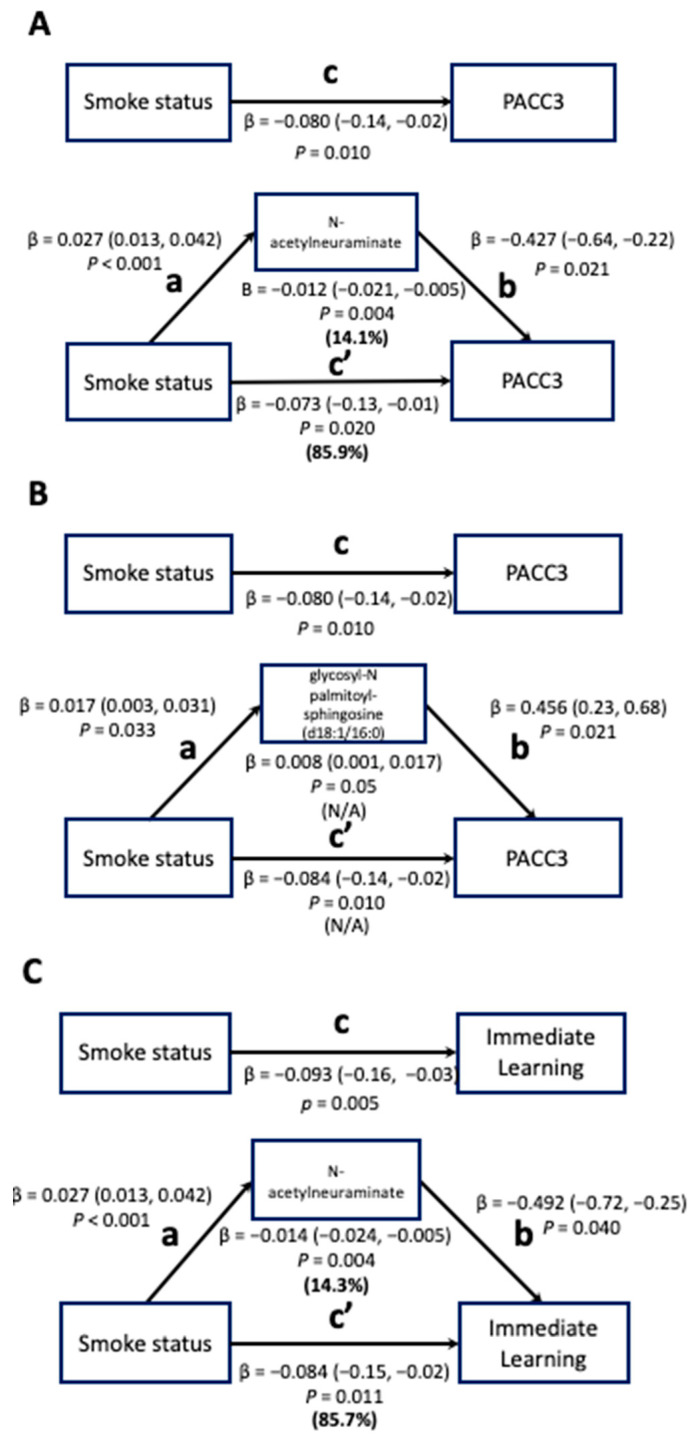
Total effect and mediation models for associations between smoking status and cognitive function mediated by metabolites (*n* = 1188). FDR-corrected *p*-values were used and are displayed for the smoking-metabolite and metabolite-cognition associations. Path c in the total-effect models represents the coefficient for the association between smoking status and PACC/Immediate Learning. Path a in the mediation models represents the coefficient for the association between smoking status and metabolites (N-acetylneuraminate (NeuAc) and glycosyl-N palmitoyl-sphingosine (d18:1/16:0) (GlcCer)) and path b in the mediation models represents the coefficient for the association between metabolites (NeuAc and GlcCer) and PACC/Immediate Learning. Using the product method, the indirect effect of metabolites (NeuAc and GlcCer) in the pathways of smoking-cognitive functions was computed (shown below the metabolite box) and path c’ in the mediation models represents the direct effect of smoking status on PACC/Immediate Learning. The proportions of the indirect effect and direct effect out of the total effect are shown in bold but could not be calculated for panel B due to the different signs between the indirect effect and the total effect.

The effect of smoking status on PACC3 in the total effect model was −0.080 (95% CI: −0.14–−0.02); 14.1% of the total effect was mediated by NeuAc in the mediation model (95% CI: −0.021–−0.005; Figure 2A). The effect of smoking status on IMM in the total effect model was −0.093 (95% CI: −0.16–−0.03; Figure 2B). NeuAc also mediated 14.3% of the total effect between smoking status and IMM in the mediation model (95% CI: −0.024–−0.005).

GlcCer mediated the relationship between smoking status and PACC3 in the mediation model (95% CI: 0.001–0.017; Figure 2C). However, the proportion of the total effect mediated by GlcCer could not be calculated because the IE and DE of GlcCer have different signs, resulting in an inconsistent mediation effect, meaning this metabolite most likely acts as a suppressor variable that indicates the presence of the mediator increases the magnitude of the DE [37].

## 4. Discussion

To the best of our knowledge, this study is the first to identify potential metabolic pathways between smoking and cognitive function. We assessed the role of CSF and plasma metabolites as mediators in this relationship in WRAP, a longitudinal observational cohort study. We showed that plasma metabolites, including NeuAc, androstenediol (3alpha, 17alpha) monosulfate (2), GlcCer, and metabolonic lactone sulfate were significantly associated with both smoking status and cognitive outcomes. Among these four plasma metabolites, NeuAc and GlcCer partially mediated the relationship between smoking status and one or more cognitive composite scores.

NeuAc is a member of the sialic acid family. Given their location and ubiquitous distribution, sialic acids can mediate or modulate a wide variety of physiological and pathological processes [38]. Our results showed that NeuAc mediated the relationships between smoking status and both PACC3 and IMM, where smoking was correlated with higher levels of NeuAc, which in turn was correlated with lower scores on both the PACC3 and IMM cognitive composites. NeuAc is the most well-known sialic acid and smoking has been shown to increase sialic acid levels. Studies have demonstrated various roles of sialic acids in the development of AD pathology [39]. For example, sialic acid–CD33 interaction can efficiently regulate microglial–resident immune cell recognition and lead to beta amyloid accumulation in the brain. CD33, one of the top-ranked AD risk genes, is highly expressed in microglia and has elevated expression in AD brains [40,41]. Elevation of sialic acid levels in the circulation has been observed in not only AD, but also in aging [42] and a wide range of AD comorbidities, such as obesity [43], diabetes [44], and cardiovascular disease [45]. One study suggested that the effects of N-acetylneuraminic acid, the predominant sialic acid, on the immune cells in the periphery were the driving force of the accelerated disease manifestations in CD4+ T cells of mice an humans [46]. Therefore, NeuAc may provide mechanistic insight into the deleterious effect of smoking on cognitive function and ADRD risk.

GlcCer is a complex sphingolipid that contains one or more sialic acids [47]. This molecule plays a role as a mediator in the relationship between smoking status and PACC3. Smoking was correlated with higher levels of GlcCer, which in turn was correlated with higher performance on the PACC3. Although counterintuitive, these results are consistent with findings that the concentrations of sphingolipid metabolites in plasma were significantly increased in smokers [48] and decreased in AD patients [49]. This implies that GlcCer could be a suppressor and mediates the relationship between smoking and cognitive function, thus exploring it further will be important in future studies.

This study was the first to explore the role of plasma and CSF metabolites as mediators in the smoking-cognitive function relationship. The results point to the role that sialic acids may play in this relationship. The limitations of this study should be considered. First, the small sample size for CSF metabolites (166 individuals) had less power to detect CSF metabolites that were associated with smoking status and that mediate the relationship between smoking and cognitive function. This should be examined in a future study with a larger sample size. Second, we did not have detailed smoking history data to allow us to more carefully examine the dose–response relationship between smoking, cognitive function, and metabolites. Third, while we adjusted for many potential confounding factors, there may be additional unobserved confounders. However, the results were similar between the reduced and fully-adjusted models, giving us confidence in the robustness of our findings. Future studies in larger, more diverse samples with longitudinal data are necessary to confirm our results and discover additional plasma and CSF metabolites that mediate the relationship between smoking and both cognitive function and ADRD.

In conclusion, our findings provide new links between previous studies that can enhance our understanding of potential biological pathways between smoking and cognitive function. Of the two significant mediators between smoking and cognitive function in our study, one, NeuAc, is a sialic acid and the other, GlcCer, contains sialic acids, providing evidence for the sialic acid associated pathway in the established smoking-cognitive function relationship. A better understanding of the biological mechanisms between smoking and cognitive function could inform future intervention studies and potentially reduce the burden of ADRD.

## Figures and Tables

**Table 1:** Baseline characteristics of participants from the WRAP sample (*n* = 1266).

Variable	Value
Age (years)	
Mean (SD)	58.5 (6.47)
Median (Min, Max)	59.0 (40.7, 75.0)
Female sex	891 (70.4%)
White race	1200 (94.8%)
College or graduate degree	777 (61.4%)
Smoking status	
Never smoker	711 (56.2%)
Former smoker	469 (37.0%)
Current smoker	86 (6.8%)
CES-D score (60 points)	
Mean (SD)	7.13 (7.16)
Median (Min, Max)	5.00 (0, 44.0)
Body Mass Index	
Underweight	8 (0.6%)
Normal	352 (27.8%)
Overweight	449 (35.5%)
Obese	457 (36.1%)
Weekly alcohol consumption	
Mean (SD)	4.25 (6.74)
Median (Min, Max)	2.00 (0, 52.5)
PACC3	
Mean (SD)	0.0160 (0.760)
Median (Min, Max)	0.0717 (−3.17, 2.55)
Immediately Learning (IMM)	
Mean (SD)	0.0145 (0.783)
Median (Min, Max)	0.0365 (−3.41, 2.36)
Delayed Recall (DEL)	
Mean (SD)	0.0155 (0.780)
Median (Min, Max)	0.109 (−3.94, 1.96)
Executive Function (EXE)	
Mean (SD)	0.00546 (0.822)
Median (Min, Max)	0.0970 (−6.42, 2.43)

Values for sex, race, education level, smoking status, and body mass index are frequencies (%). Smoking status: never smoker, former smoker and current smoker are defined as participants who have never smoked cigarettes, have smoked cigarettes but not in the past month, and have smoked cigarettes in the past month, respectively. CES-D score is the sum of 15 categories, each with a scale of 0–4. Body mass index (BMI): underweight: BMI < 18.5 kg/m^2^, normal: 18.5 kg/m^2^ <= BMI < 25.0 kg/m^2^, overweight: 25.0 kg/m^2^ <= BMI < 30.0 kg/m^2^ and obese: BMI >= 30.0 kg/m^2^. Weekly alcohol consumption: one drink being defined as a 12 oz. beer, a 4 oz. glass of wine, or one shot (1.25 oz.) of liquor.

**Table 2:** Linear mixed model analysis of the association between smoking status and cognitive function in the WRAP sample (*n* = 1266).

Predictors	PACC3				Immediate Learning				Delayed Recall				Executive Function			
	Beta	SE	95% CI	*p*	Beta	SE	95% CI	*p*	Beta	SE	95% CI	*p*	Beta	SE	95% CI	*p*
Intercept	−1.098	0.095	−1.285–−0.910	<0.001	−0.955	0.101	−1.154–−0.756	<0.001	−0.881	0.102	−1.082–−0.681	<0.001	−1.059	0.104	−1.262–−0.855	<0.001
Female	0.529	0.039	0.452–0.605	<0.001	0.443	0.041	0.362–0.523	<0.001	0.388	0.041	0.307–0.469	<0.001	0.354	0.042	0.271–0.437	<0.001
White race	0.400	0.083	0.236–0.563	<0.001	0.353	0.088	0.181–0.525	<0.001	0.327	0.089	0.153–0.501	<0.001	0.552	0.092	0.372–0.732	<0.001
College or graduate degree	0.375	0.037	0.302–0.448	<0.001	0.358	0.039	0.281–0.434	<0.001	0.358	0.040	0.280–0.435	<0.001	0.266	0.041	0.186–0.346	<0.001
CES-D	−0.005	0.001	−0.007–−0.002	<0.001	−0.005	0.001	−0.008–−0.002	0.001	−0.004	0.001	−0.007–−0.001	0.005	−0.007	0.001	−0.010–−0.005	<0.001
BMI (underweight)	−0.238	0.093	−0.421–−0.055	0.011	−0.157	0.109	−0.372–0.057	0.151	−0.090	0.109	−0.303–0.124	0.41	−0.170	0.090	−0.346–0.007	0.06
BMI (overweight)	0.004	0.024	−0.042–0.051	0.855	0.011	0.028	−0.043–0.065	0.691	−0.001	0.027	−0.054–0.053	0.984	0.004	0.023	−0.041–0.050	0.858
BMI (obese)	0.032	0.029	−0.025–0.089	0.27	0.039	0.033	−0.026–0.104	0.244	0.026	0.033	−0.039–0.090	0.441	−0.027	0.029	−0.083–0.030	0.358
Alcohol Weekly Consumption	0.005	0.002	0.002–0.008	0.002	0.005	0.002	0.002–0.009	0.004	0.004	0.002	0.000–0.007	0.034	0.006	0.001	0.003–0.009	<0.001
Former smoker	0.053	0.038	−0.021–0.127	0.159	0.025	0.039	−0.052–0.102	0.524	0.057	0.040	−0.021–0.135	0.151	0.024	0.041	−0.056–0.105	0.557
Current smoker	−0.383	0.073	−0.526–−0.240	<0.001	−0.360	0.077	−0.510–−0.210	<0.001	−0.360	0.077	−0.512–−0.209	<0.001	−0.270	0.078	−0.424–−0.117	0.001
Age	−0.047	0.003	−0.052–−0.041	<0.001	−0.039	0.003	−0.045–−0.033	<0.001	−0.035	0.003	−0.041–−0.029	<0.001	−0.060	0.003	−0.066–−0.054	<0.001
Age^2^	−0.001	0.000	−0.001–−0.001	<0.001	−0.001	0.000	−0.001–−0.000	<0.001	−0.001	0.000	−0.001–−0.000	<0.001	−0.001	0.000	−0.001–−0.001	<0.001
Practice Effect	0.082	0.008	0.067–0.098	<0.001	0.124	0.009	0.107–0.141	<0.001	0.117	0.009	0.100–0.134	<0.001	0.074	0.008	0.058–0.091	<0.001
Random Effects																
ICC	0.78				0.71				0.73				0.83			
*N* (family)	1007				1007				1007				1007			
*N* (Individual)	1266				1266				1266				1266			
Observations	4680				4680				4680				4680			
Marginal R^2^/Conditional R^2^	0.298/0.844				0.216/0.773				0.194/0.779				0.283/0.881			

Abbreviations: SE, standard error, ICC, intraclass correlation coefficient, correlation among observations within the sibling group and repeated measures within an individual; DBID, WRAP coded database ID number. Marginal R^2^ indicates the variance explained only by fixed effects and conditional R^2^ indicates the variance explained by both fixed and random effects.

**Table 3:** Association test for smoking-associated metabolites and cognitive function (*n* = 1188).

Cognitive Function	Plasma Metabolite	Estimates	95% CI	*p*	FDR
PACC3	glycosyl-N palmitoyl-sphingosine (d18:1/16:0)	0.456	0.680–0.233	<0.001	0.021
	N-acetylneuraminate	−0.427	−0.636–−0.219	<0.001	0.021
IMM	N-acetylneuraminate	−0.429	−0.733–−0.251	<0.001	0.040
EXE	metabolonic lactone sulfate	0.177	0.091–0.263	<0.001	0.035
	androstenediol (3alpha, 17alpha) monosulfate (2)	0.135	0.066–0.204	<0.001	0.037

Each model was adjusted for sex, race, education level, visit number (practice effect), linear and quadratic terms for age, CES-D, BMI, and weekly alcohol consumption. Random intercepts for family and individual and a random slope for age were included in the models to account for correlation between siblings and in repeated measures within an individual.

## Data Availability

The data underlying this article can be requested through the WRAP Application for Resources link on the following website: https://wrap.wisc.edu/data-requests-2/ (released on May 2020).

## Supplementary Materials

The following supporting information can be downloaded at: https://www.mdpi.com/article/10.3390/metabo13111154/s1, Figure S1: Correlations between cognitive composite scores; Figure S2: Manhattan plot of the associations between smoking status and CSF metabolites; Figure S3: Manhattan plot of the associations between smoking status and plasma metabolites; Table S1: Linear mixed model analysis of the association between smoking status and cognitive function in the WRAP sample (Full and reduced models, *n* = 1266); Table S2: Associations between smoking status and metabolomics; Table S3: Associations between metabolites and cognitive function.
