# Supplementary material for: Assessing the Biological Mechanisms Linking Smoking Behavior and Cognitive Function: A Mediation Analysis of Untargeted Metabolomics
Source: Metabolites. 2023 Nov 16;13(11):1154. doi: 10.3390/metabo13111154 (PMC10673384; doi:10.3390/metabo13111154)
Supplement: Supplementary file 1 [file metabolites-13-01154-s001.zip › Supplementary Figures_Final.pdf]

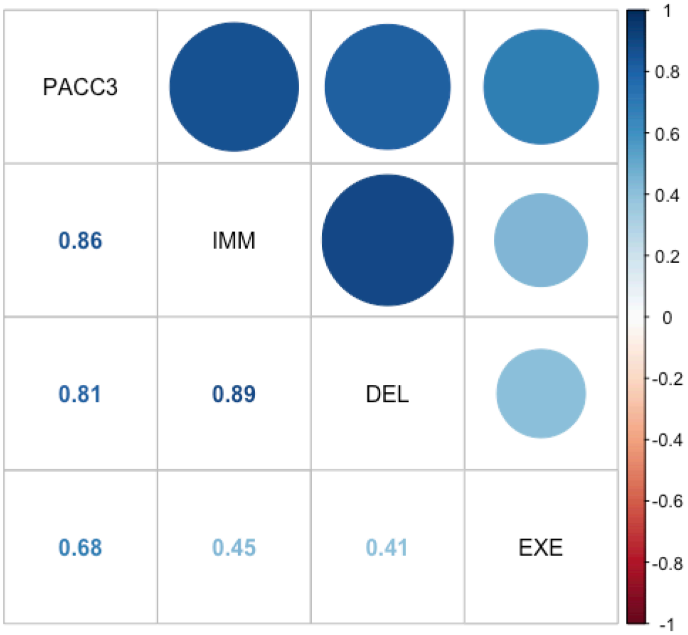

Supplementary Figure S1. Correlations between cognitive composite scores. Blue circles indicate positive correlations while red circles indicate negative correlations. The size of the circle corresponds to the degree of correlation. PACC3: 3-test Preclinical Alzheimer Cognitive Composite; IMM: Immediate Learning; DEL: Delayed Recall; EXE: Executive Function.

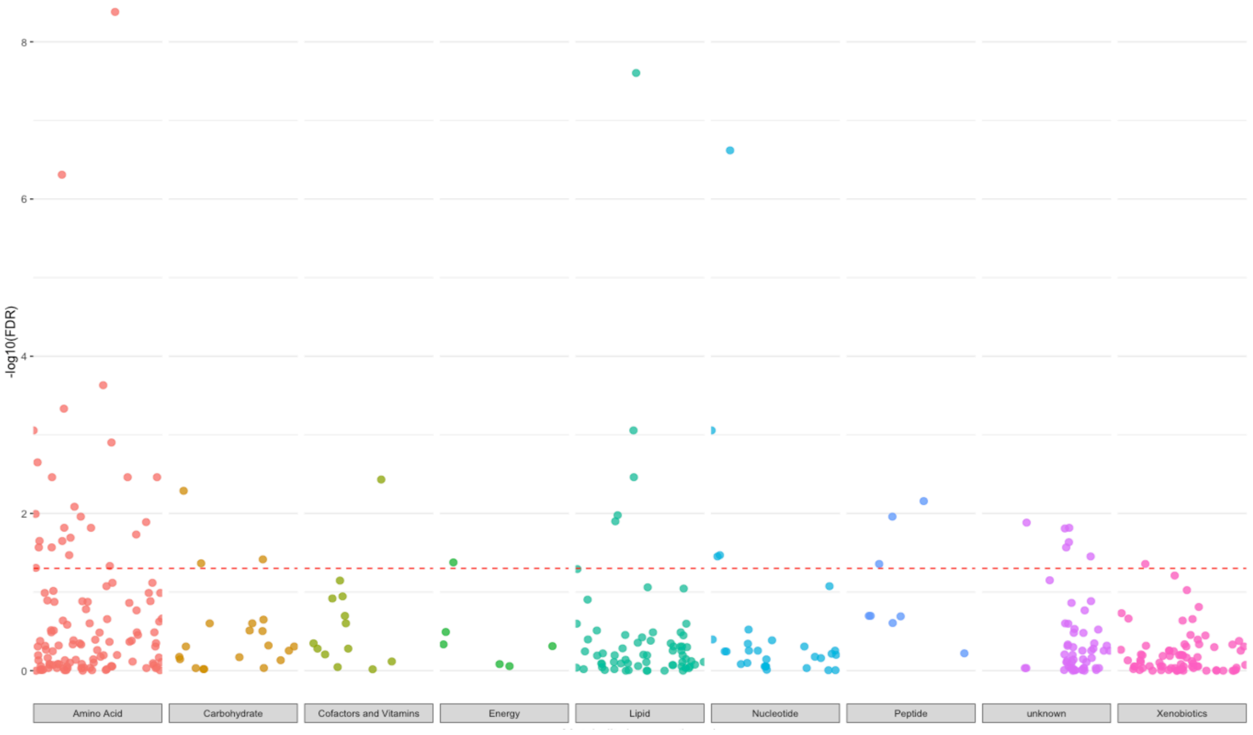

Supplementary Figure S2. Manhattan plot of the associations between smoking status and CSF metabolites. Each linear mixed model included a random intercept for the individual and a random slope for age. The outcome was a metabolite and smoking status, sex, race, education level, practice effect, linear and quadratic terms for age, CES-D, BMI, and weekly alcohol consumption were adjusted for. Each dot represents a metabolite. The y-axis is the -$\log_{10}$ of the FDR-adjusted p-value for the association between smoking status and the CSF metabolite. The metabolites are categorized by super pathway, displayed on the x-axis (metabolites in the unknown category are novel metabolites that are not in existing databases).

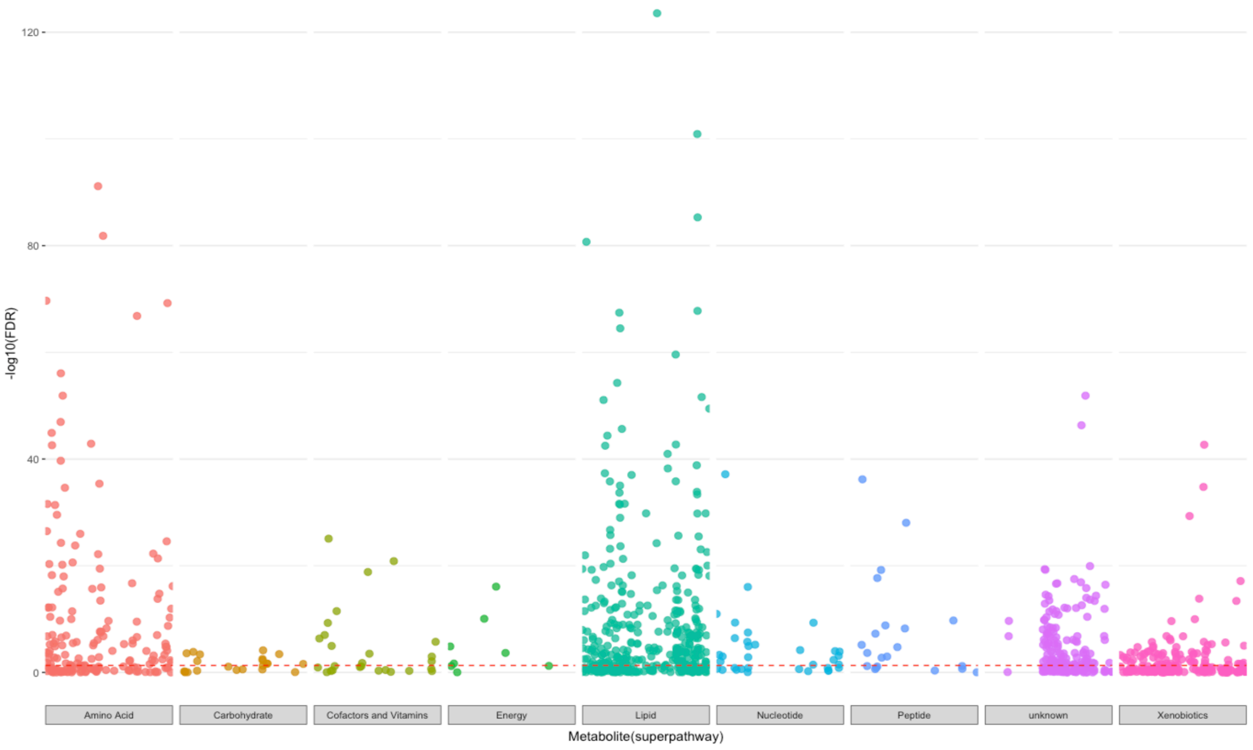

Supplementary Figure S3. Manhattan plot of the associations between smoking status and plasma metabolites. Each linear mixed model included a random intercept for family and individual and a random slope for age. The outcome was a metabolite and smoking status, sex, race, education level, practice effect, linear and quadratic terms for age, CES-D, BMI, and weekly alcohol consumption were adjusted for. Each dot represents a metabolite. The y-axis is the -$\log_{10}$ of the FDR-adjusted p-value for the association between smoking status and the plasma metabolite. The metabolites are categorized by super pathway, displayed on the x-axis (metabolites in the unknown category are novel metabolites that are not in existing databases).
